# Optogenetic inhibition of the limbic corticothalamic circuit does not alter spontaneous oscillatory activity, auditory-evoked oscillations, and deviant detection

**DOI:** 10.1038/s41598-024-63036-5

**Published:** 2024-06-07

**Authors:** Irene Gonzalez-Burgos, Miguel Valencia, Roger Redondo, Philipp Janz

**Affiliations:** 1 Roche Pharma Research and Early Development, Neuroscience and Rare Diseases, Roche Innovation Center Basel, F. Hoffmann-La Roche Ltd, Grenzacherstrasse 124, 4070 Basel, Switzerland; 2 Program of Biomedical Engineering, Universidad de Navarra, CIMA, Avenida Pío XII 55, 31080 Pamplona, Spain; 3 IdiSNA, Navarra Institute for Health Research, 31080 Pamplona, Spain

**Keywords:** Neuroscience, Neural circuits, Sensory processing

## Abstract

Aberrant neuronal circuit dynamics are at the core of complex neuropsychiatric disorders, such as schizophrenia (SZ). Clinical assessment of the integrity of neuronal circuits in SZ has consistently described aberrant resting-state gamma oscillatory activity, decreased auditory-evoked gamma responses, and abnormal mismatch responses. We hypothesized that corticothalamic circuit manipulation could recapitulate SZ circuit phenotypes in rodent models. In this study, we optogenetically inhibited the mediodorsal thalamus-to-prefrontal cortex (MDT-to-PFC) or the PFC-to-MDT projection in rats and assessed circuit function through electrophysiological readouts. We found that MDT–PFC perturbation could not recapitulate SZ-linked phenotypes such as broadband gamma disruption, altered evoked oscillatory activity, and diminished mismatch negativity responses. Therefore, the induced functional impairment of the MDT–PFC pathways cannot account for the oscillatory abnormalities described in SZ.

## Introduction

Aberrant neuronal circuit dynamics are at the core of complex neuropsychiatric disorders, such as schizophrenia (SZ). SZ symptoms are classified into positive (i.e. delusions and hallucinations), negative (i.e. anhedonia and social withdrawal), and cognitive symptoms (i.e. deficits in working memory, executive function, and processing speed)^1^. This SZ symptomatology is driven by neuronal circuit dysfunction. For example, dopamine in the mesolimbic system has been speculated to be responsible for positive symptoms, dopamine in the mesocortical pathway for negative symptoms, and glutamate in the prefrontal circuitry for cognitive symptoms^2^.

Functional impairment of neuronal communication in SZ has been evidenced through electrophysiological and functional MRI (fMRI) studies that consistently show aberrant synchronization of certain neural oscillations^1,3,4^.

Indeed, robust endophenotypes have been reported in the clinic. A consistent finding in SZ is aberrant resting-state gamma-band oscillatory activity (30–100 Hz)^5^. Additional circuit mechanisms that appear impaired when probed in SZ patients are auditory-driven oscillations and context-dependent auditory processing (i.e. mismatch negativity [MMN])^6,7^. Extensive data exists demonstrating deficits in 40 Hz auditory steady-state responses (ASSR) in SZ patients^8–10^. Entrainment to other oscillatory frequencies is also reduced as evidenced by auditory chirp-evoked potential (chirp-AEP) studies^11^. Furthermore, reduced mismatch responses are among one of the best-characterized endophenotypes in SZ and psychosis^12,13^. But what underlying circuit physiology could account for these functional biomarkers in SZ endophenotypes?

Both changes in anatomical connectivity as well as in neuronal functional activity deficits play a role in SZ. Structurally, magnetic resonance imaging (MRI) and positron emission tomography studies have shown that SZ patients present gray matter deficits, white matter volume changes, aberrant axonal microstructure, and altered myelination^1,3,14^. On the other hand, fMRI studies report hypoconnectivity within thalamic-sensonsory-motor-cortices, and hyperconnectivity in thalamic-prefrontal-striatal-cerebellar networks^15–19^. Magnetoencephalography studies show increased thalamocortical gamma connectivity during visual processing in SZ^20^. The prefrontal cortex (PFC), thalamus, and temporal lobe present distorted activation while cognitive actions are performed by SZ patients^2,21^. Patients and individuals at risk of SZ present a decreased correlation between PFC and mediodorsal thalamus (MDT) under resting conditions^21^. Recently, increased thalamocortical connectivity was proposed as a central circuit mechanism for altered sensory processing during psychosis and psychedelic states^22^. Overall, these studies highlight the importance of aberrant activity in the corticothalamic circuit in SZ.

In this study, employing optogenetic tools that allow for the exploration of disease-relevant projections, we set out to determine whether the previously documented hypoconnectivity of the thalamocortical MDT–PFC pathway could underlie key psychiatric endophenotypes observed in humans^6,7,9–13,23–26^ and translational animal models^27–33^. The PFC, a brain region crucial for cognitive function, is densely innervated by the thalamus, most prominently the MDT. Contrary to other thalamic nuclei, the MDT mainly receives input from the PFC^21^. Accordingly, we optogenetically inhibited the MDT-to-PFC and the PFC-to-MDT projections in freely moving rats and assessed circuit function by studying: (i) spontaneous oscillatory activity and coherence across brain areas, (ii) auditory-evoked oscillations using chirp-modulated tones, and (iii) MMN-like responses using simple tones. By inhibiting the MDT–PFC projection, we predicted an increase in spontaneous gamma oscillations accompanied by an impairment in synchronization to evoked gamma. The MDT is one of the primary thalamic nuclei that regulate the PFC. In turn, the PFC is a region known to participate in higher-order cognitive processing, in which gamma oscillatory activity is a tool. Therefore, when inhibiting the regulation that MDT plays over the PFC, we hypothesized that the gamma activity of the PFC would be aberrant and dysfunctional. Furthermore, such optogenetic manipulation would be expected to impair the MMN response, as a measure of prediction error and adaptation.

## Results

### Activation of the inhibitory Jaws2 opsin inhibits neuronal function both ex vivo and in vivo

**Figure 1:**
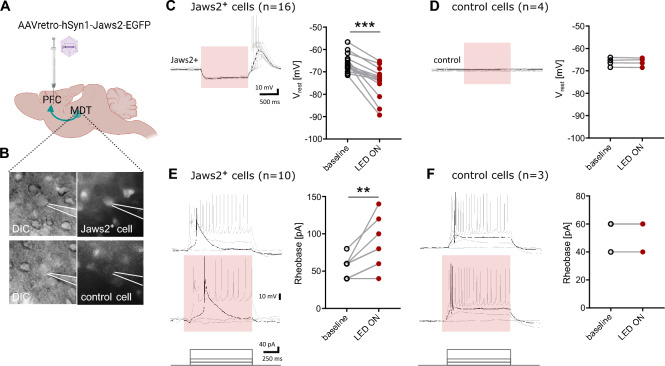
Jaws2-activation successfully inhibits neuronal activity ex vivo. (**A**) For the MDT-to-PFC, the AAVretro-hSyn1-Jaws2-EGFP was injected into the prefrontal cortex (PFC) from where it retrogradely traveled toward the soma of PFC-projecting neurons of the mediodorsal thalamus (MDT). (**B**) In acute brain slices, whole-cell recordings were performed from Jaws2-expressing and non-expressing neurons. (**C**) LED pulses depolarized Jaws2^+^ cells (n = 16) and led to rebound hyperpolarization upon release. (**D**) LED pulses did not affect the membrane potential of control cells (n = 4). (**E**) LED pulses altered the firing rate and action potential threshold (Rheobase) of Jaws2^+^ cells (n = 10). (**F**) LED pulses did not alter the firing rate or action potential threshold of control cells (n = 3). Data tested with Wilcoxon matched-pairs test; **p < 0.01, ***p < 0.001.

Firstly, we corroborated in acute slices that Jaws2-expression effectively reduces neuronal excitability (Fig. 1). Indeed, activating Jaws2 through 630 nm red LED illumination drives strong hyperpolarization of Jaws2^+^ cells (n = 16) with rebound excitation upon release from photoinhibition (Fig. 1C) as compared to non-expressing control cells (n = 4) (Fig. 1D). Additionally, Jaws2-mediated hyperpolarization increases the threshold for neurons to fire action potentials (n = 10) (Fig. 1E) as compared to control cells (n = 3) (Fig. 1F). These results confirm that Jaws2 is appropriate for driving strong neuronal inhibition in our experiments.

**Figure 2:**
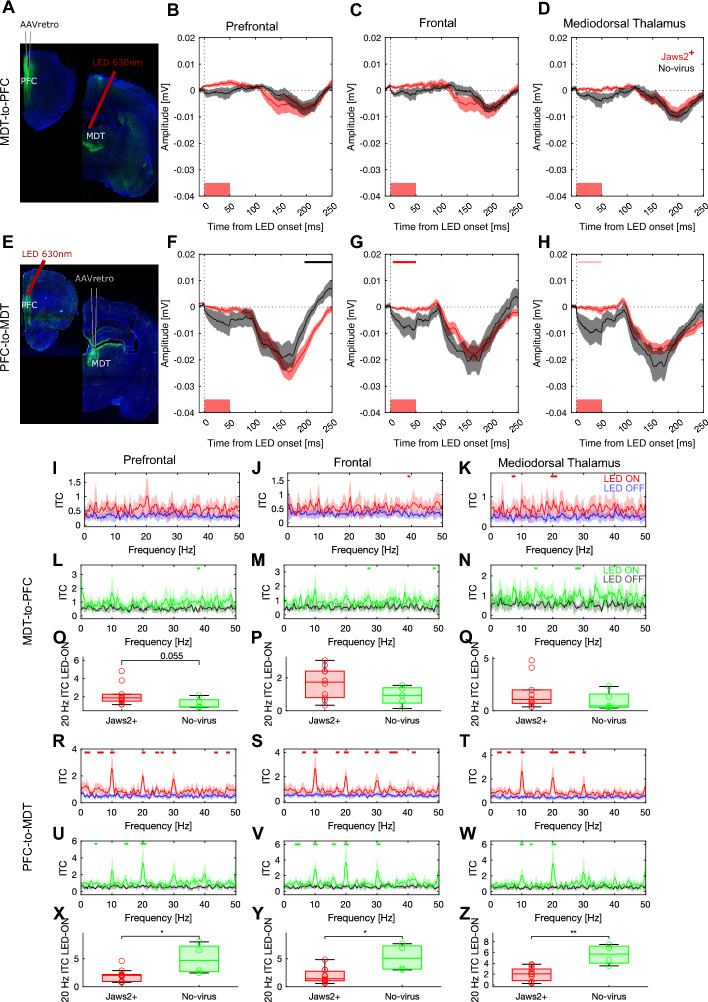
Jaws2-activation successfully inhibits neuronal activity in vivo. (**A**) Post-mortem histological confirmation of expression of Jaws2 in the MDT after being injected in the PFC in the MDT-to-PFC animals. Animals were implanted with a screw electrode over the frontal cortex and an LFP electrode in the MDT. Also over the MDT, the optic fiber was implanted. (**B**–**D**) In vivo LED stimulation causes a double deflection; in animals expressing Jaws2, the LED pulse partially inhibits the first deflection at ~ 50 ms. (**E**) Post-mortem histological confirmation of expression of Jaws2 in the PFC after being injected in the MDT in the PFC-to-MDT animals; in which the optic fiber was implanted in the PFC. (**F–H**) In vivo LED stimulation causes a double deflection; in animals expressing Jaws2, the LED pulse inhibits the first deflection at 0–50 ms. Red boxes at the bottom of the graphs indicate LED-on stimulus duration (0–50 ms). Solid lines indicate the average and shaded areas describe the standard error of the mean. Top horizontal red lines indicate significant clusters identified by paired cluster-based permutation analysis. LED 10 Hz pulse stimulation induces intertrial coherence (ITC) peaks at 10 Hz and its harmonics in the MDT-to-PFC cohort in (**I–K**) Jaws2^+^ animals (blue: LED off; red: LED on) and (**L**–**N**) control animals (black: LED off; green: LED on). (**O**–**Q**) ITC peak at 20 Hz in Jaws2^+^ (red) and no-virus control animals (green). Wilcoxon signed-rank test was performed and in cases in which results are significant they are shown (*p < 0.05, **p < 0.01). LED 10 Hz pulse stimulation induces intertrial coherence (ITC) peaks at 10 Hz and its harmonics in the PFC-to-MDT cohort in (**R–T**) Jaws2^+^ animals (blue: LED off; red: LED on) and (**U**–**W**) control animals (black: LED off; green: LED on). (**X–Z**) ITC peak at 20 Hz in Jaws2^+^ (red) and no-virus control animals (green). Wilcoxon signed-rank test was performed and in cases in which results are significant they are shown (*p < 0.05, **p < 0.01).

Next, we proceeded to corroborate whether it would also be functional in vivo (Fig. 2). Posthoc histology demonstrated sufficient Jaws2 expression in targeted brain areas (Supplementary Figs. 1 and 2) of all virally-injected animals. Of note, PFC injections resulted mainly in Jaws2-expression in cells near the midline of the mediodorsal thalamic region, including the medial part of the mediodorsal thalamic nucleus, intermediodorsal thalamic nucleus, and paraventricular nucleus. MDT targeted injections resulted in the expression of Jaws2 in deep layers of the PFC. A pilot study was conducted to probe the optogenetic effects on electrophysiological readouts in vivo, using 50 ms long LED pulses (interstimulus interval: 400 ± 100 ms) in both Jaws2-expressing (N = 21) and control (N = 10) animals. We observed that the presentation of 630 nm pulses elicits changes in electric field potentials independently of the opsin expression (Fig. 2B–D, F–H). The pulse-evoked response is characterized by a late deflection at ~ 100–250 ms in both the Jaws2^+^ and no-virus groups. This suggests the induction of visual-evoked responses by the LED pulses, which are independent of opsin expression. The magnitude of this late deflection depends on the anatomical localization of the optic fiber, being more pronounced in animals where the optic fiber was placed above the PFC (N = 12) (Fig. 2E–H) in comparison to those in which it was implanted over the MDT (N = 9) (Fig. 2A–D).

Moreover, in animals with the optic fiber implanted over the PFC, there is an earlier deflection at ~ 0–50 ms in the no-virus control animals (N = 12) (Fig. 2F–H). Importantly, in animals with Jaws2^+^ PFC-to-MDT neurons, the early deflection is inhibited during the presentation of the pulse (~ 0–50 ms) in the frontal cortex (Fig. 2G; CBPT *p* < 0.05) and tends to also be inhibited in the MDT (Fig. 2H; CBPT *p* < 0.1). In the PFC, the Jaws2^+^ animals show a similar effect (Fig. 2F). Furthermore, although less pronounced, in the MDT-to-PFC cohort there is also a tendency for reduced field potential deflections in the ~ 0–50 ms time window for Jaws2^+^ animals (Fig. 2B–D).

To further support that Jaws2-mediated photoinhibition in vivo was successful, we assessed changes in phase coherence of optically-induced oscillations between rats that express the inhibitory opsin Jaws2 and rats that do not. LED photostimulation induces a phase-resetting in all animals (Fig. 2I–Z). This is reflected as a 10 Hz intertrial coherence (ITC) peak and its subsequent harmonics at 20, 30, and 40 Hz. This phase resetting is very pronounced in animals in which the optic fiber was placed in the PFC (Fig. 2R–W), and less so in animals being stimulated in the MDT (Fig. 2I–N), likely because of the larger distance of the fiber to the retina.

When comparing ITC of Jaws2-expressing animals to controls, the ITC induced at 20 Hz differed significantly between groups in the PFC-to-MDT cohort. In the case of the MDT-to-PFC cohort, the ITC at 20 Hz in the PFC tends to be enhanced (Fig. 2O). On the contrary, the presence of Jaws2^+^ in the PFC-to-MDT cohort significantly reduced ITC at 20 Hz in all brain regions studied (Fig. 2X–Z).

Taken together, these results suggest a silencing of neuronal activity via Jaws2 in vivo, resulting in a reduced first deflection response, but simultaneously demonstrates that there is a Jaws2-independent pulse-evoked activity that needs to be considered for further analysis and the interpretation of our results.

### Photoinhibition of the MDT–PFC circuit does not affect spontaneous oscillatory activity

**Figure 3:**
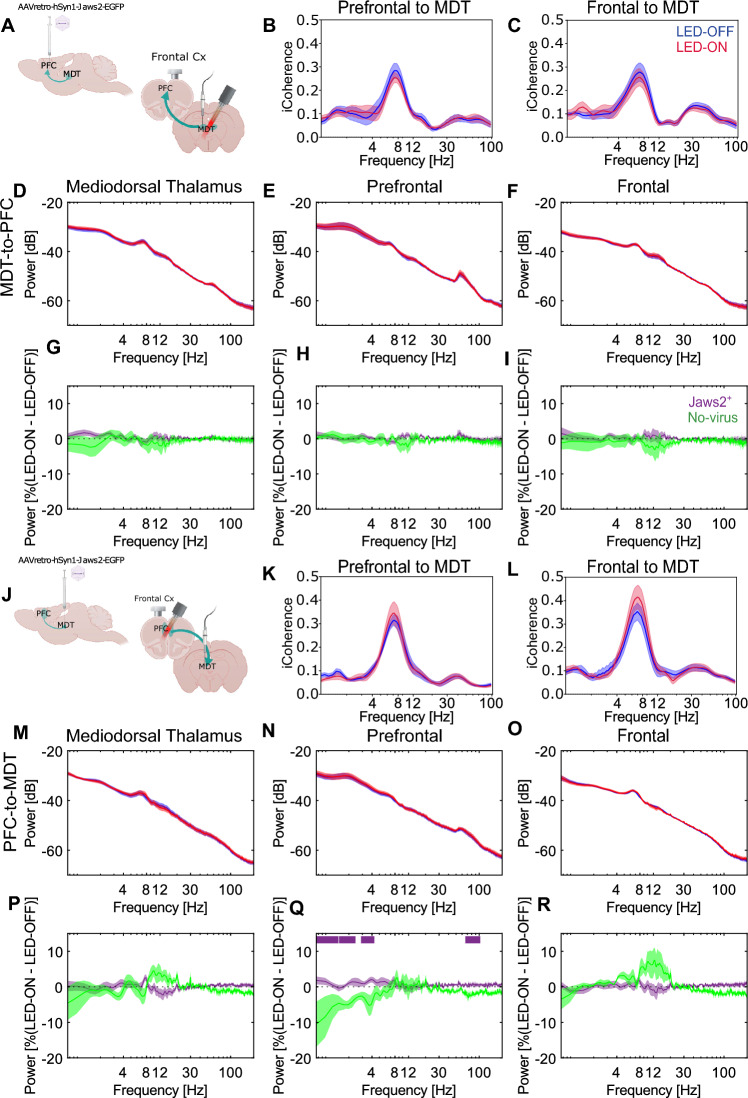
Photoinhibition of the reciprocal PFC-MDT connection has limited effects on the resting-state oscillatory activity. (**A**) Schematic of experimental configuration for the MDT-to-PFC photoinhibition. Imaginary coherence between (**B**) the PFC and MDT (N = 8), and (**C**) the Frontal cortex and MDT (N = 8) in the LED-off (blue) and LED-on condition (red; 10 Hz: 20 ms ON—80 ms OFF) during MDT-to-PFC inhibition. Power-spectral density estimation in the (**D**) MDT (N = 9), (**E**) PFC (N = 9), and (**F**) Frontal cortex (N = 9). Average of the within-animal differences between the LED-on and LED-off conditions for Jaws2-positive (purple, N = 9) and no-virus controls (green, N = 5) in the (**G**) MDT, (**H**) PFC, and (**I**) Frontal cortex. (**J**) Schematic of experimental configuration for the PFC-to-MDT photoinhibition. Imaginary coherence between (**K**) the PFC and MDT (N = 11), and (**L**) the Frontal cortex and MDT (N = 11) in the LED-off (blue) and LED-on condition (red; 10 Hz: 20 ms ON—80 ms OFF) during PFC-to-MDT inhibition. Power-spectral density estimation in the (**M**) MDT (N = 11), (**N**) PFC (N = 11), and (**O**) Frontal cortex (N = 11). Average of the within-animal differences between the LED-on and LED-off conditions for Jaws2-positive (purple, N = 11) and no-virus controls (green, N = 4) in the (**P**) MDT, (**Q**) PFC, and (**R**) Frontal cortex. Solid lines indicate the average and shaded areas describe the standard error of the mean. Top horizontal purple lines indicate significant clusters identified by paired cluster-based permutation analysis.

To infer basic brain circuit function, we analyzed the spectral composition of spontaneous oscillatory activity during alternating sessions of photoinhibition at 10 Hz (20 ms on) and no-photoinhibition (Supplementary Fig. 3A) as well as alterations in functional coupling within the limbic circuit by calculating the imaginary coherence between the MDT and the frontal cortex or the PFC. Inhibition of the MDT-to-PFC neurons (Fig. 3A) through 10 Hz photoactivation of Jaws2 does not lead to changes in coherence (Fig. 3B, C) and the spectral composition of brain signals (Fig. 3D–I). This coherence and oscillatory activity is also unaltered by inhibiting the reciprocal PFC-to-MDT (Fig. 3J) neurons (Fig. 3K–R). Of note, the 10 Hz photostimulation in no-virus control animals (N = 5) leads to a decrease in slow frequencies (~ 0–4 Hz) and in high-gamma (~ 70–100 Hz) in the PFC when inhibiting the PFC-to-MDT projection (Fig. 3Q). No other significant differences between the Jaws2^+^ and the no-virus controls were found. Hence, there are no effects on ongoing oscillatory activity by inhibiting the PFC-MDT projections.

### Photoinhibition of MDT–PFC circuit does not alter auditory evoked oscillations

**Figure 4:**
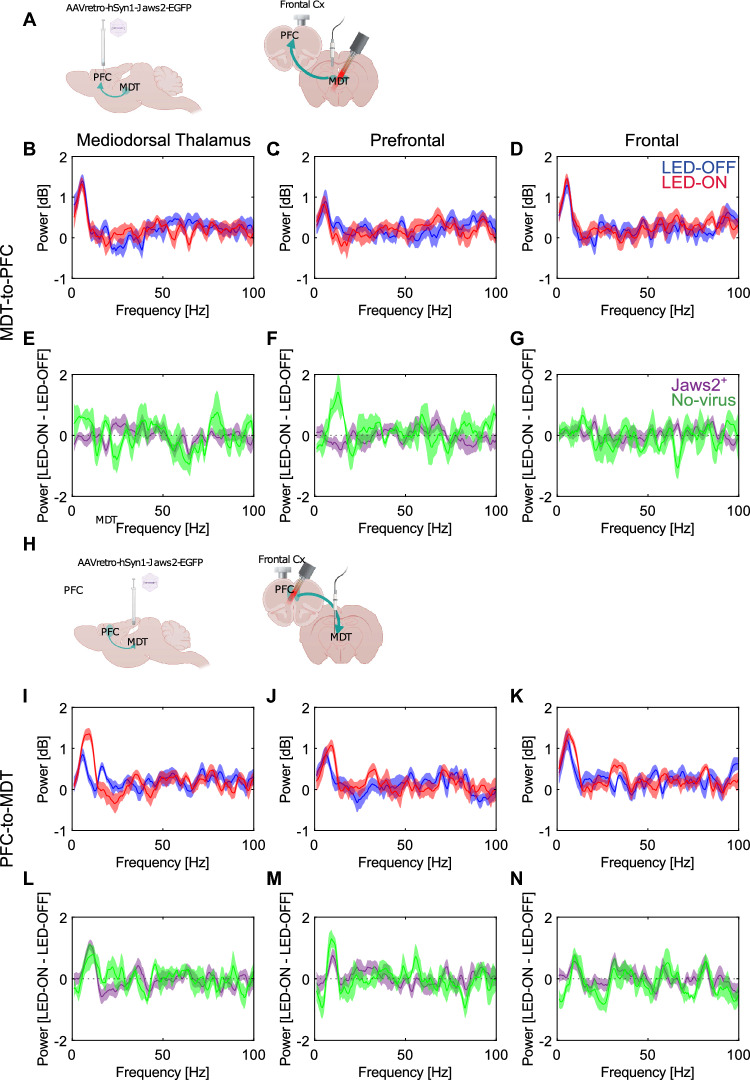
Photoinhibition of the reciprocal PFC-MDT connection does not affect chirp-evoked oscillatory activity. (**A**) Schematic of experimental configuration for the MDT-to-PFC photoinhibition. Spectral power density plots showing the evoked activity (normalized for pre-stimulus baseline) across the frequency range of the chirp-stimulus (1–100 Hz) in the LED-off (blue) and LED-on (red) conditions in the (**B**) MDT (N = 10), (**C**) PFC (N = 10) and (**D**) Frontal cortex (N = 10), and within animal differences between the LED-on and LED-off conditions for Jaws2-positive (purple) and no-virus controls (green, N = 4) in the (**E**) MDT, (**F**) PFC, and (**G**) Frontal cortex during MDT-to-PFC inhibition. (**H**) Schematic of experimental configuration for the PFC-to-MDT photoinhibition. Spectral power density of evoked activity (normalized for pre-stimulus baseline) across the frequency range of the chirp-stimulus (1–100 Hz) in the LED-off (blue) and LED-on (red) conditions in the (**I**) MDT (N = 12), (**J**) PFC (N = 10), and (**K**) Frontal cortex (N = 11), and within animal differences between the LED-on and LED-off conditions for Jaws2-positive (purple) and no-virus controls (green, N = 5) in the (**L**) MDT, (**M**) PFC, and (**N**) Frontal cortex during PFC-to-MDT inhibition. Solid lines indicate the average and shaded areas describe the standard error of the mean. The absence of top horizontal lines indicates no significant clusters were identified by paired cluster-based permutation analysis.

We used chirp-AEP to test the capacity of neurons to synchronize their activity with a sensory stimulus. We found that photoinhibition induced by 2-s-long continuous pulses while the chirp stimulus is being presented (Supplementary Fig. 3B) slightly changed the chirp-AEP in the PFC-to-MDT (Fig. 4H–N). The power of the chirp-AEP is unchanged when silencing the MDT-to-PFC neurons (Fig. 4A–G). In contrast, inhibition of the PFC-to-MDT neurons tended to increase Chirp-AEP power at ~ 10 Hz in the MDT (Fig. 4I; N = 11) as well as the PFC and frontal cortex (Fig. 4J–K; N = 11).

Interestingly, the increase in the power of auditory-evoked oscillation at ~ 10 Hz was similar for Jaws2^+^ PFC-to-MDT animals and the corresponding no-virus control animals (N = 4). Therefore, this change in power does not appear to be due to Jaws2-mediated inhibition.

Of note, a similar increase in ~ 10 Hz oscillatory power was also found in the prefrontal cortex of no-virus controls (N = 5) of the MDT-to-PFC cohort (Fig. 4F). In contrast, MDT-to-PFC Jaws2-expressing animals did not show this increase in auditory-evoked oscillatory power, which supports the inhibitory action of Jaws2 in vivo during the presentation of the chirp.

### Photoinhibition of the PFC-MDT circuit does not impair local mismatch responses

To assess the role of the MDT–PFC projection in cognitive circuit function, we elicited mismatch responses using an advanced auditory oddball paradigm. In humans, the corresponding readout is the MMN response, which is produced when an unexpected sensory input (“deviant” stimulus) conflicts with a memory trace established by repeated presentation of a “standard” stimulus^34^. In our study, we compared the responses elicited without photoinhibition to the ones obtained when accompanying the tones with 50 ms continuous pulses (Supplementary Fig. 2C). MMN-like responses were found in the MDT-to-PFC and PFC-to-MDT animals in both the Jaws2^+^ and no-virus animals (Supplementary Fig. 4). Evoked responses to the deviant stimuli presented higher amplitude than the responses to the control tone, while amplitude of the response to the standard stimulus was diminished.

**Figure 5:**
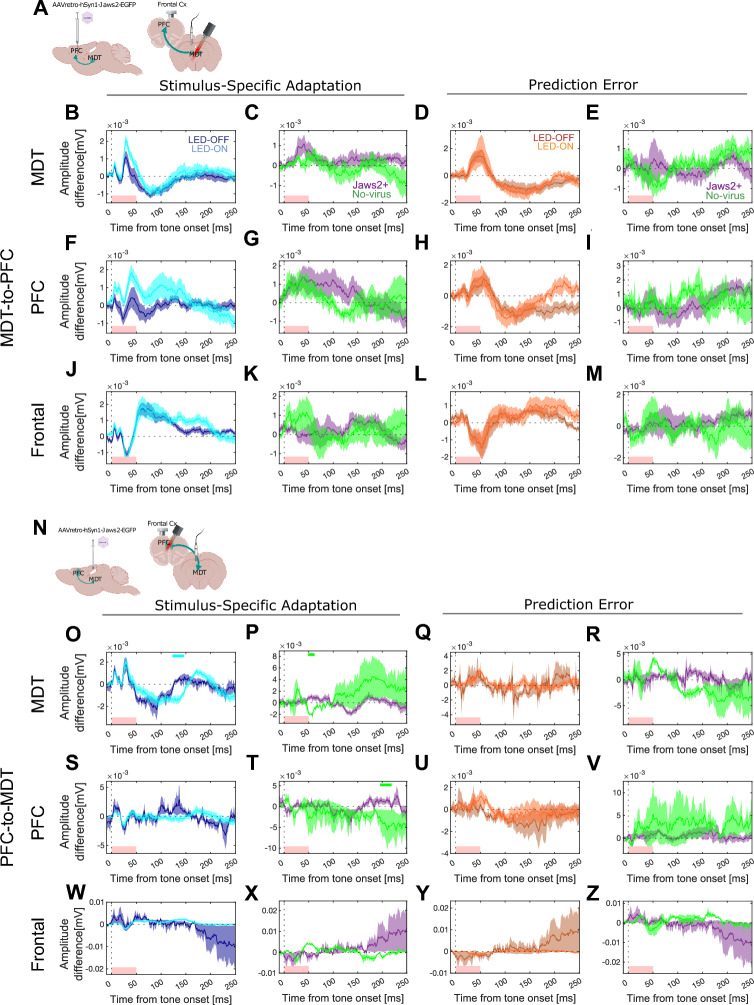
Photoinhibition of the reciprocal PFC-MDT connection does not interfere with mismatch responses. (**A**) Schematic of the MDT-to-PFC experiment. Average ERP waveforms for the MDT-to-PFC stimulus-specific adaptation in LED-on (cyan) and LED-off (navy) conditions (starting from the left, first column), difference in adaptation between LED-on and LED-off in Jaws2^+^ animals (purple) and no-virus controls (green) (second column), prediction error in LED-on (orange) and LED-off (brown) conditions (third column), and difference in prediction error between LED-on and LED-off in Jaws2^+^ animals (purple) and no-virus controls (green) (fourth column) in the MDT (**B**–**E**), PFC (**F**–**I**), and Frontal (**J**–**M**). (**N**) Schematic of the PFC-to-MDT experiment. Average ERP waveforms in MDT (**O**–**R**), PFC (**S**–**V**), and Frontal cortex (**W**–**Z**). Red boxes at the bottom of the graphs indicate LED-on stimulus duration (0–50 ms). Solid lines indicate the average and shaded areas describe the standard error of the mean. The absence of top horizontal lines indicates no significant clusters were identified by paired cluster-based permutation analysis.

**Figure 6:**
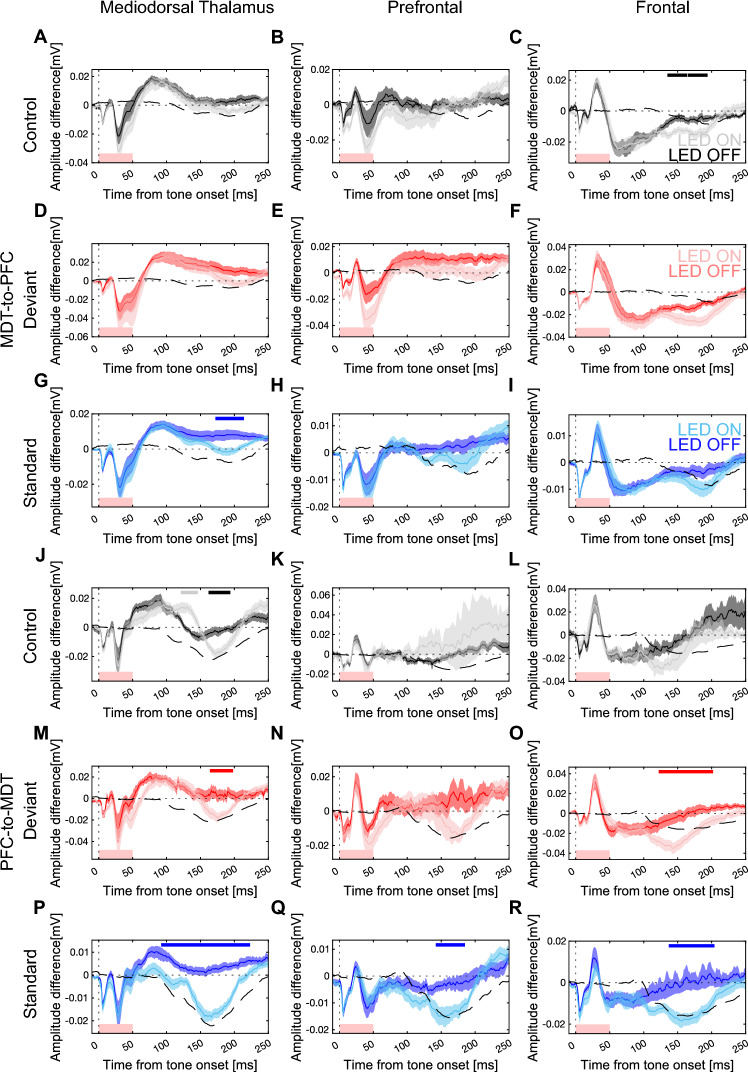
Photoinhibition of the reciprocal PFC-MDT connection affects the late component of the evoked responses but not the mismatch response. Evoked responses to the control (black, **A**–**C**, **J–L**); to the deviant (red, **D–F**, **M**–**O**); and the standard tone (blue, **G–I**, **P–R**) in LED-on (light) and LED-off (dark) conditions while inhibiting the MDT-to-PFC projection (**A**–**I**) and the PFC-to-MDT (**J**–**R**) in the MDT (left), PFC (center), and Frontal cortex (right). Solid lines indicate the average and shaded areas describe the standard error of the mean. Top horizontal lines indicate clusters identified by paired cluster-based permutation analysis. Dashed black lines indicate the average evoked response to LED photostimulation in the absence of auditory stimulation. Red boxes at the bottom indicate LED-on stimulus duration (0–50 ms).

Differential waveforms relative to the control tone reveal the contribution of prediction error and adaptation components to the overall mismatch responses. In this experiment, the inhibition of the PFC-MDT projections showed no changes in the prediction error or adaptation in any of the areas studied (Fig. 5). Importantly, no differences between the responses in the Jaws2-positive and the ones in no-virus controls were observed. In fact, when comparing the waveforms within the three conditions (control, deviant, and standard) in the LED on and off conditions, the only differences appear during the late presumably visually-evoked deflection at ~ 100–250 ms (Fig. 6). This indicates that the inhibition being elicited in the MDT–PFC circuitry in this experiment does not impair the MMN response.

## Discussion

This work aimed to understand the contribution of the MDT–PFC pathway to endophenotypes of psychiatric disorders. After confirming that optogenetic inhibition occurred in the targeted projection, we found no changes in translational circuit function readouts. Photoinhibition of the MDT–PFC circuit did not replicate the main endophenotypes repeatedly reported in SZ^6,7,9–13,23–26^. To our knowledge, this is the first study to assess the effect of optogenetic inhibition of the MDT–PFC circuitry on spontaneous and auditory-evoked oscillations as well as MMN-like responses.

We validated the tool in vivo and ex vivo*,* with slice recordings that showed that red LED drives strong hyperpolarization of Jaws2^+^ cells, which is accompanied by rebound excitation upon release from photoinhibition^35^. Furthermore, Jaws2-mediated hyperpolarization increases the threshold for neurons to fire action potentials. Collectively, this data confirms that the construct being expressed in neurons has an inhibitory effect driven by red LED in slice recordings.

When analyzing the responses to 630 nm LED pulses in awake freely-moving animals, we found that illumination can elicit a response even when the opsin is not present. The response consists of a small early deflection (~ 0–50 ms) and a large late deflection (~ 100–250 ms). We hypothesize that this response may be visually-evoked. Supporting this hypothesis is the fact that the more anterior the placement of the fiber optic (i.e., closer to the retina of the animal), the more prominent the response. As such, the LED-induced responses are more prominent in the PFC-to-MDT cohort than in the MDT-to-PFC cohort. The effects of illumination on neuronal tissue are a well-documented confounding factor^36^. Danskin et al*.*^37^ found that red illumination delivered deep into mouse brain tissue through an optical fiber activates opsins in the retina and causes behavioral artifacts. Our results support the notion that LED-driven effects are an inevitable confound in optogenetic studies and therefore highlight the need to account for this effect by including the appropriate controls. In our case, we have included animals that do not express the opsin as controls for between-subject comparison.

Compared to the necessary opsin-negative controls, the data shows that the early deflection of the response (~ 0–50 ms) to the photostimulation in Jaws2^+^ animals is decreased in the frontal cortex and the MDT of the PFC-to-MDT cohort. A similar tendency is present in the PFC, as well as in the MDT-to-PFC cohort.

Additionally, we have found that the induced phase-locking by LED at 20 Hz is altered due to the expression of Jaws2^+^ . We hypothesized that differences should be more present in the 20 Hz band, although the underlying stimulation was at 10 Hz, since the evoked deflection is biphasic (e.g. see Fig. 2E–H), mapping approximately to the 20 Hz band. Phase resetting has been previously shown for sensory-evoked oscillations^38,39^. In this context, a stimulus can ‘reset’ ongoing oscillations rather than adding to ongoing oscillations, a phenomenon that can be revealed with ITC but not necessarily with ‘classical’ power spectral density analyses. Since the induced phase-locking by LED at 20 Hz is altered due to the expression of Jaws2^+^ , a functional opsin-driven effect in vivo confirms Jaws2-mediated neuronal inhibition.

Nevertheless, when comparing the effects between the two different conditions (i.e. MDT-to-PFC and PFC-to-MDT) there are seemingly opposing directions on ITC. Although there is not a sufficient explanation for this, one may hypothesize that because the circuit motifs of the PFC-to-MDT and the MDT-to-PFC pathways are different (e.g. MDT drives feedforward inhibition on mPFC pyramidal cells via interneurons, promoting string paired-pulse depression^40^ which is different to PFC-projections to MDT eliciting paired-pulse facilitation^41^), inhibition of one or the other projection may manifest differently on physiological readouts. Nevertheless, given the limited understanding of these circuits, we want to avoid further speculations.

Having established the utility of the chosen optogenetic paradigm, we used it to manipulate corticothalamic circuit function. In this work, we have shown that photoinhibition of the PFC-to-MDT or the MDT-to-PFC projection, according to our experimental parameters, has no effects on the ongoing spontaneous oscillatory activity. The only change is that, compared to no-virus control animals in which there is a decrease in slow frequencies between the LED-on and LED-off condition, Jaws2^+^ animals present no changes in slow frequencies in the MDT during photoinhibition of the PFC-to-MDT.

Notably, we found that spontaneous gamma-band oscillatory activity is unchanged when inhibiting the corticothalamic circuit. In contrast, alterations in spontaneous gamma-band oscillatory activity in SZ patients have been extensively documented^42^. Since this manipulation of the corticothalamic MDT–PFC circuit does not recapitulate this key SZ endophenotype, our results indicate that this circuit does not seem to play a key role in the enhancement of gamma-band oscillations. However, with the present results and due to the limitations of the techniques employed, one cannot disregard that other thalamocortical circuits could be involved.

As in the case of the ongoing oscillatory activity, imaginary coherence between the PFC and the MDT is not affected by the inhibition of this direct projection. Similarly, other studies using pharmacological models of SZ based on *N*-methyl-D-aspartate receptor (NMDAR) antagonism also found no alterations in coherence between these two nuclei^27,43^.

Of note, we included a 10 Hz inhibitory paradigm to explore if analysis of power spectral densities and coherence of spontaneous oscillations could be a sensitive measure to pick up the biological effects of Jaws2 engagement in vivo. Based on the previously presented data ex vivo, we acknowledge that this stimulation paradigm may also drive rebound excitation in vivo. In fact, we considered that if enough neurons would exert rebound excitation, this may manifest as a population spike in the EEG signal and an associated peak in the PSD around the stimulation frequency. Such a signal could have served as an easily accessible surrogate marker for Jaws2 activation. We interpret the lack of such a rebound deflection as an indication that the number of inhibited cells was too low to be evident in the population activity. This is reasonable considering that with local injections only a fraction of projections cells will be retrogradely infected and express the inhibitory construct. In contrast, direct evidence for inhibition necessitates the detection of photosensitive units of either spontaneously active neurons or stimulus/task-driven active neurons, which was not applicable in our experimental configuration and led us to capitalize on other measures previously described.

In contrast, for the auditory paradigms, the LED stimulation was adjusted to match the presentation and duration of the auditory stimulus (i.e. to restrict inhibition when integration of the stimulus is initiated or ongoing). As such, the LED pulse lasted 50 ms to match the length of the stimuli during the MMN paradigm and 2 s to match the length of the stimulus during the Chirp paradigm.

Concerning evoked oscillations, the inhibition of the MDT–PFC projection has no effect on chirp-driven oscillatory activity. The 2-s-long LED pulses during the chirp-AEP paradigm in the PFC-to-MDT cohort lead to an enhancement of ~ 10 Hz oscillations in both Jaws2^+^ and no-virus control animals. Of note, during this paradigm, the photostimulation was continuous during the 2 s that the chirp-AEP was presented, so enhancement at 10 Hz is not directly related to the visually-evoked ‘artifact’. A plausible explanation may be an interaction between auditory and visual processing that ultimately leads to an enhancement of the auditory responses. This interaction between auditory-evoked oscillations and the visual stimulation from the fiber is supported by previous studies that have reported an impact of visual and auditory salient cues on ASSR^44^. All in all, these results do not recapitulate what has been described in SZ patients who show impairments in evoked gamma synchronization^11^, especially in the 40-Hz ASSR paradigm^45,46^. Specifically, in the high-gamma range, decreases in activity have been observed during higher-order cognitive tasks^47^.

Lastly, attenuated MMN responses are a consistent finding in SZ, independent of disease stage or characteristics of the stimuli^48^. In SZ, it was shown that mainly the prediction error component of the MMN is diminished^49^. In our rodent study, the photoinhibition of the MDT–PFC reciprocal circuit does not affect the prediction error or the adaption component. Our results indicate that this corticothalamic circuit might not be not responsible for the MMN endophenotype found in SZ. The only changes between the photoinhibition conditions observed are at time points in which the LED induces a pronounced deflection. Therefore, we hypothesize that these detected changes are due to the effect of a visually-evoked response, but that the optogenetic inhibition itself had no major impact on the generation of the MMN-like response.

A notable observation is that the prediction error diminishes when implanting the optic fiber in the PFC. Previous studies have reported that lesions to the PFC impair prediction error in humans^50^. We believe the observed diminished prediction error in our study may be due to the damage elicited by the implantation of the optic fiber in regions that have been proposed to be key in higher-order sensory processing.

In this study, we have identified two confounding factors of relevance for all optogenetic studies: (i) the LED meant to activate the opsin elicits a response independent of the presence of the construct, and (ii) the damage elicited by implanting the optic fiber leads to aberrant higher-order cognitive processing. To further study the possible damage, future studies could apply chemogenetics or minimally-invasive optogenetic protocols that allow the silencing of circuit nodes without the confound of arguably larger lesions caused by optic fibers. In addition, specifically for our study, we have proved the construct to be functional. However, questions remain on whether the magnitude of the inhibition of the circuit is sufficient to elicit clear changes on the network level. Given that with local delivery of AAV only a subgroup of cells express the opsin, we cannot exclude that the number of inhibited cells is not enough to clearly disrupt circuit function. In conclusion, the key SZ phenotypes (aberrant spontaneous gamma oscillations, reduced induced evoked gamma activity, and MMN deficits) have not been recreated with the current paradigm used to inhibit the PFC-MDT corticothalamic circuit used in this study.

## Methods

### Animals

Experiments were conducted in 38 adult male Sprague Dawley rats (RRID:RGD 734,476, average age: 12 weeks, average weight: 350 g) purchased from Charles River, Germany. Rats were kept in a 12 h light/dark cycle at room temperature. Sample sizes were based on previous experiments to provide sufficient statistical power with the expected effect sizes of our readouts-of-interest. Food and water were provided ad libitum. All animals were sacrificed with appropriate anesthesia agents and euthanasia methods (see sections “Slice recordings” and “Perfusion and histological verification” for details). All procedures were approved by the Federal Food Safety and Veterinary Office of Switzerland and conducted in strict adherence to the Swiss Federal Ordinance on Animal Protection and Welfare, as well as according to the Association for Assessment and Accreditation of Laboratory Animal Care International rules. The study is reported in accordance with ARRIVE guidelines.

### Viral injections

Rats were deeply anesthetized with 4% isoflurane for 5 min in an incubation chamber and received a subcutaneous injection of buprenorphine (0.1 mg/kg) for further analgesic treatment. Analgesia was selected according to official guidelines at the time of the experiments. Throughout the surgery, isoflurane levels were kept at 2–3% using an inhalation mask.

The AAVretro-hSyn1-Jaws_KGC_EGFP_ER2-WPRE-hGHp(A) was synthesized by the Viral Vector Facility of the University of Zürich. A Hamilton injection syringe was lowered 0.5 mm past the indicated depth. Then the syringe was raised to the desired depth and 1 µL virus was injected at a rate of 100 nL/min. Animals were randomly assigned to one of the two experimental cohorts, and within each cohort to the viral vector or the no-vector control group. The first experimental cohort, MDT-to-PFC, was injected in the bilateral PFC with coordinates (relative to bregma; all dorsoventral (DV) coordinates measured from cortical surface): anteroposterior (AP) = 2.5 mm; mediolateral (ML) =  ± 0.6 mm; DV = − 3.6 mm with viral vector (N = 13) or saline (N = 5). The second experimental cohort, PFC-to-MDT, was injected in the bilateral MDT with coordinates: AP = − 3.3 mm; ML = ± 0.8 mm; and DV = − 5.0 mm with viral vector (N = 12) or saline (N = 5). Optic fibers were implanted in the MDT for the MDT-to-PFC group (AP = − 3.3 mm; ML = 3 mm; and DV = − 3.5 mm at a 33° angle) and in the PFC for the PFC-to-MDT group (AP = 2.5 mm; ML = 1.4 mm; and DV = − 4.3 mm at a 10° angle). Note that with the angle with which the fibers were implanted optic stimulation reached both hemispheres, so optogenetic manipulation occurred bilaterally.

### Slice recordings

For terminal experiments, rats (N = 4) were deeply anesthetized with 2.0% isoflurane (Abbott, Cham, CH) and decapitated. The brain was quickly removed and transferred to the slicing chamber filled with ice-cold *N*-Methyl-d-glucamin (NMDG) solution containing (in mM) 110 NMDG, 3 KCl, 1.1 NaH2PO4, 25 NaHCO3, 103.02 HCl, 25 d-glucose, 10 l-ascorbic acid, 3 Pyruvic acid, 0.5 CaCl2 * 2H2O and 10 MgCl2 * 6H2O. The brain was sliced into 350 µm thick frontal sections using a VT1000S vibratome (Leica, Wetzlar, GER). Acute slices were recovered in NMDG solution for 15 min at 35 °C and then transferred to ACSF containing (in mM) 124 NaCl, 2.5 KCl, 1 NaH2PO4, 25 NaHCO3, 20 d-glucose, 2 CaCl2 * 2H2O and 1 MgCl2 * 6H2O adjusted to 305–310 mOsm. Patch-clamp whole-cell recordings were performed at room temperature and at a perfusion rate of 1.5 ml/min.

For patch-clamp experiments, both Jaws2-expressing and non-expressing cells were identified and recorded in whole-cell mode within the same slice. Before approaching the cells with the patch pipette, a fiber optic (diameter: 200 µm; 0.66NA) coupled to a 630 nm LED (Plexon Inc., Dallas, Texas, USA) was placed in close proximity to the region of recording. The patch pipette (GC150F-10; Harvard Apparatus) contained a solution of (in mM) 145 KMeSO4, 10 Hepes, 10 NaCl, 10 EGTA, 5 MgATP, 0.5 Na2ATP, 1 CaCl2 * 2H2O and 1 MgCl2 * 6H2O (adjusted to 290 mOsm and pH 7.2) and had a resistance 2.5–3.5 MΩ. Using positive pressure, cells were visually approached. By applying negative pressure, a gigaseal was formed with the targeted cell. Then, the cell membrane was opened and protocols were started 5 min after opening to allow for a sufficient exchange of intracellular solution and normalization of cell physiology. The signal was preamplified with a CV-7B electrode holder (Axon Instruments, Molecular Devices, San Jose, California, US) and again amplified and digitized with a MultiClamp 700B (Axon Instruments) using 100 × AC membrane potential (200 mV/mV) mode, a Bessel filter at 2.4 kHz, and alternating current at 1 Hz. Signals were recorded and analyzed with Clampex software (Axon Instruments). Only cells for which leak currents did not exceed ± 100 pA and for which the axial resistance did not show changes higher than 20% were included for analysis. To infer Jaws2-mediated hyperpolarization, the membrane potential was measured before and during a 1 s-long pulse of 630 nm LED (power set to 200 mA) activating Jaws2. Intrinsic excitability was tested using stepwise current injections (from 10 to max. 200 pA in 20 pA current steps every 10 s; duration: 1 s) with or without 630 nm LED (onset 10 ms before current injection; total duration: 1 s; power 200 mA). Photoinhibition (LED-ON) and without photoinhibition (LED-OFF) conditions were randomized.

### Electrode implantation

In the same surgery as the viral injection, animals (N = 34) were implanted with Innovative Neurophysiology 16 channel movable array, positioned to target the left MDT (AP = − 3.3 mm; ML = -0.8 mm; and DV = − 4.0 mm). A tungsten electrode to measure local field potentials (LFP) was placed in the left prefrontal cortex (AP = mm; ML = mm; and DV = mm) and a stainless steel screw was placed above the frontal cortex (AP = − 2.5 mm; ML = 1.2 mm) for cumulative surface EEG measurements from both hemispheres. Two additional screws were placed as reference and ground above the cerebellum (AP = − 10 mm, ML = ± 2 mm). The implant was fixed to the skull with dental cement (Paladur®, Kulzer) and secured with UV-curing resin (Lukafix, Cat. No. D1351305; Lukadent GmbH, Schwieberdingen, GER). Post-operative analgesia (0.1 mg/kg Buprenorphine s.c. once daily) was performed for two additional days to minimize post-surgical pain. After the surgery animals were single housed in IVC rat cages (Tecniplast; Buguggiate, ITA) to prevent damage to implants. Standard environmental enrichment for implanted animals (material for nest building and gnawing) was provided. Animals were checked once daily to monitor for wound healing and signs of pain.

### Electrophysiological recordings, acoustic stimulation, and LED presentation

Electrophysiological data was collected with an OpenEphys (www.open-ephys.com) system, and animals (N = 34) were video-tracked with an infra-red USB camera (Ailipu Technology, Shenzhen, CHN) and Bonsai software 2.5.1 (www.bonsai-rx.org) as described in Janz et al*.*^27^. LED pulses (630 nm wavelength; power adjusted to 200 mA) and acoustic stimuli (calibrated to 75–80 dB SPL) were presented using a TDT RZ6-A-P1 system and RPvdsEx software (Tucker-Davis Technologies; Alachua, Florida, USA). The measured LED output power at the fiber tip was 550 uW and applied to all in vivo experiments. Three different experimental sessions were performed on five different days. All experimental sessions began with 15-min open-field freely-moving sessions without any auditory or optical stimulation. The spontaneous-oscillatory activity experimental sessions consisted of 25-min recording comprised of 5-min blocks in which animals were presented with 10 Hz LED pulses (20 ms on; 80 ms off) alternated with 5-min intervals in which no photoinhibition occurred (Supplementary Fig. 1A). In the end, two 5-min photoinhibition blocks were compared against three alternating 5-min no-photoinhibition blocks.

The chirp-AEP experimental sessions consisted of 30 min of 300 presentations of chirp stimulus^51^ for which at random half were accompanied by a 2-s LED pulse (Supplementary Fig. 1B). This allowed for within-animal comparison of the chirp-AEP with photoinhibition versus no-photoinhibition. Chirp stimuli consisted of tones modulated in amplitude from 1 to 100 Hz (carrier frequency: 5 kHz; duration: 2 s, inter-stimulus interval: 4 s, jitter: 10 ms). The 1–100 Hz diagonal band was considered for analysis.

The MMN sessions consisted of 60 min of pure tones presented in an oddball paradigm (Supplementary Fig. 1C). We used 5 and 7 kHz tones (duration: 50 ms, inter-stimulus interval: 400 ms, jitter:10 ms). Oddballs were presented with a 10% probability to elicit mismatch responses. Tones were arranged either in an “ascending oddball”—the deviant tone (10%) has a higher frequency than the standard (90%), “descending oddball”—the deviant tone (10%) has a lower frequency than the standard (90%), or “many-standards” sequence—the deviant tone (10%) appears within a sequence of equiprobable tones. In this context, the tone of interest (7 kHz) will therefore be the deviant in the ascending, the standard in the descending, and the control in the many-standards. Thus, for the ‘many-standards’ paradigm, the ERP trace obtained when averaging the tone of interest (7 kHz) constitutes the control, and the rest of the equiprobable tones are discarded from further analysis. Individual sequences were repeated two times to account for putative habituation effects over time. This paradigm was done in two separate sessions: one day without photoinhibition, and another day with 50 ms LED pulses synchronized to the auditory tones. The 50 ms LED pulses were presented in a third separate session following the oddball paradigm scheme but without any auditory stimulation.

### Perfusion and histological verification

Rats were deeply anesthetized with ketamine (75 mg/kg) plus xylazine (11 mg/kg) and intracardially perfused with phosphate-buffered (0.1 M PBS) 4% paraformaldehyde (PFA) solution. The brains were resected and placed in PFA for post-fixation for 24 h. Following post-fixation, the brains were transferred to 30% sucrose PBS solution for another 24 h and shock-frozen afterward. Brains were cut with a cryotome into 50 µm-thick coronal sections. Sections were mounted on SuperFrost® (Menzel Gläser) slides and the endogenous green fluorescence protein (GFP) signal was qualitatively assessed to confirm viral expression.

### Data processing and analysis

We used a custom MATLAB-based workflow for all data operations. Raw data were downsampled to 1000 Hz, and artifacts were identified and excluded from the analysis. Video-tracking data was processed and synchronized to the electrophysiological data, allowing for the classification into two distinct behavioral states. Segments in which the center-of-mass of the animal moved at a speed superior to 2.5 cm/s for at least 1 s were classified as ‘active. The complementary segments were classified as ‘inactive. The ‘inactive’ segments, used for the analysis, included both quiet wakefulness as well as sleep states. Qualitative assessment of data per session was performed, and suboptimal recordings were excluded from further analysis.

For estimations of phase-locking, openfield sessions were segmented into 2-s-long signals. For the LED-ON, the beginning of each segment was determined by an LED pulse onset, and thus each segment contained 20 LED pulses. The sessions without photoinhibition were segmented at random. Then, the intertrial coherence (ITC) between the segments in each condition was estimated by calculating the Discrete Fourier Transform and extracting the phase. The ITC was then corrected per number of segments (trials) as per Sanchez-Carpintero et al.^52^.

PSD was calculated using the Welch method. EEG signal was divided into 2 s-long time series with an 80% overlap, and, for each series, the DFT was calculated for frequencies between 0.2 and 200 Hz logarithmically distributed. Imaginary coherence was calculated between the PFC and MDT electrodes. To quantify chirp-AEP responses, we analyzed the evoked activity by calculating the spectral power of the average field potential during the auditory chirp stimulation (normalized to the pre-stimulus baseline) and extracting the 1–100 Hz diagonal band. For analysis of auditory-evoked potentials, average field potentials were calculated in a − 50 to 250 ms time window relative to tone onset. For analysis, we compared average responses of 7 kHz tones between contexts: as a deviant (in the ascending sequence), standard (descending sequence), and control tone (many-standards sequence). The prediction error results from the difference between the waveform response to deviant and the response to control, and the stimulus-specific adaptation results from subtracting the response of control minus the response of standard (details in Janz et al*.*^27^). We confined our analysis to the inactive state, controlling for the putative effects of the behavioral state on ERPs.

### Statistical testing

Slice data were statistically tested with Prism 8 software (GraphPad, San Diego, California, USA), performing Wilcoxon matched-pairs test (significance level set to *p* < 0.05). Statistical testing for electrophysiological readouts was performed with a paired cluster-based permutation test (CBPT) using custom-made scripts in MATLAB. CBPT deals with the multiple comparisons problem and allows the identification of significant clusters in continuous data, e.g., in the time or frequency domain^53–55^. Here, CBPT was carried out by performing multiple paired t-tests (two-tailed, significance level set to *p* < 0.05) of the continuous data, followed by testing against permuted data (N = 1000 permutations, *p* < 0.05). For identified clusters, we report the cluster size and *p*-values. Of note, the numeric values of the reported cluster sizes can only be considered approximations^53^.

### Supplementary Information

Supplementary Figures.

## Data Availability

The datasets generated during and/or analyzed during the current study are available from the corresponding author upon reasonable request.
