# Supplementary material for: Optogenetic inhibition of the limbic corticothalamic circuit does not alter spontaneous oscillatory activity, auditory-evoked oscillations, and deviant detection
Source: Sci Rep. 2024 Jun 7;14:13114. doi: 10.1038/s41598-024-63036-5 (PMC11161607; doi:10.1038/s41598-024-63036-5)
Supplement: Supplementary file 1 — Supplementary Figures. [file 41598_2024_63036_MOESM1_ESM.pdf]

Title

Optogenetic inhibition of the limbic corticothalamic circuit does not alter spontaneous oscillatory activity, auditory-evoked oscillations, and deviant detection.

Authors

Irene Gonzalez-Burgos[abc] – igonzalezb@unav.es

Miguel Valencia[bc] – mvustarroz@unav.es

Roger Redondo[a*] – roger.redondo@roche.com

Philipp Janz[a*] – philipp.janz@roche.com

Affiliations

a. Roche Pharma Research and Early Development, Neuroscience and Rare Diseases, Roche Innovation Center Basel, F. Hoffmann-La Roche Ltd, Grenzacherstrasse 124, 4070 Basel, Switzerland
b. Universidad de Navarra, CIMA, Program of Neuroscience, 31080, Pamplona, Spain.
c. IdiSNA, Navarra Institute for Health Research, 31080, Pamplona, Spain.

Supplementary Figures

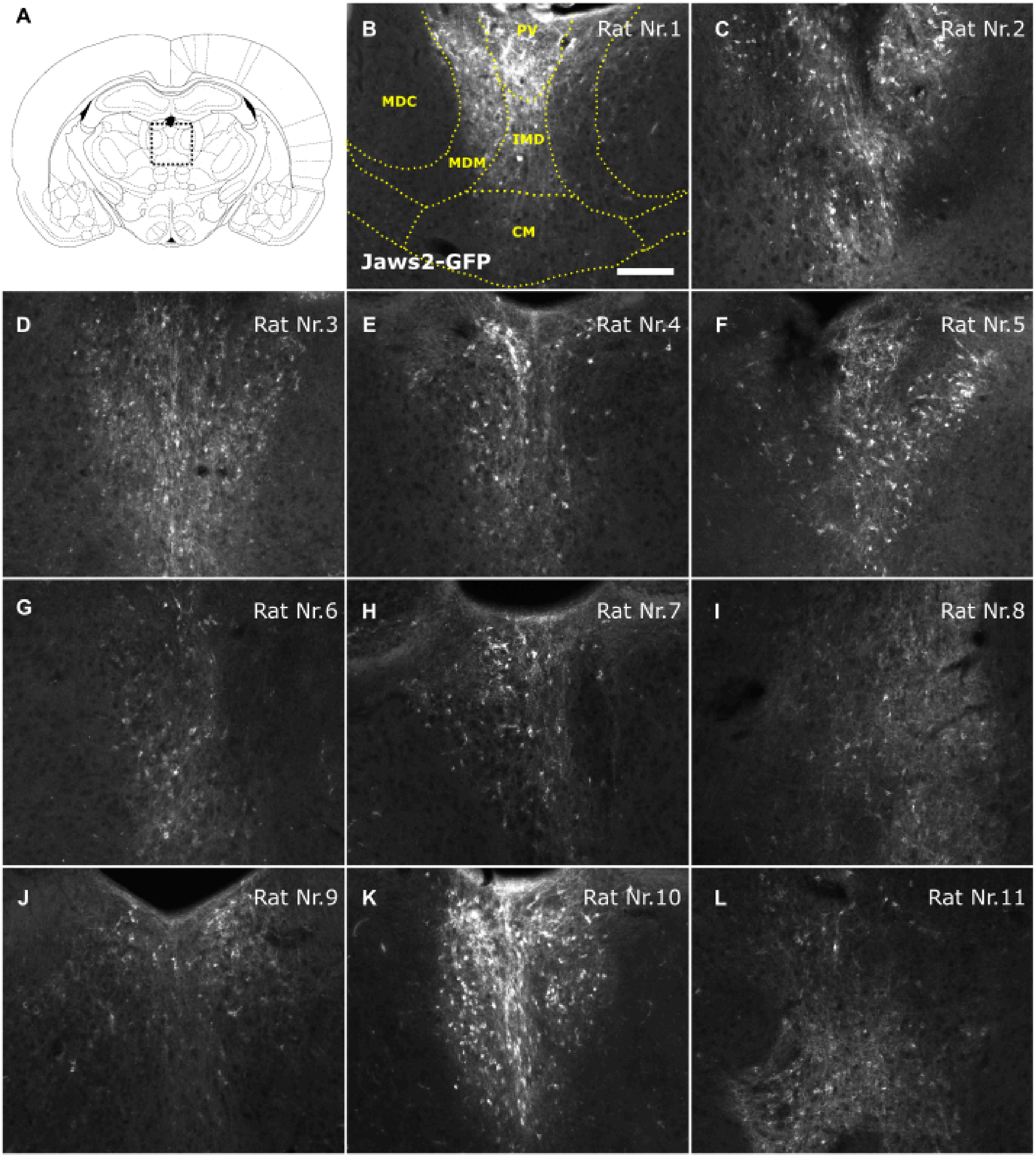

Supplementary Figure 1. Histological verification of the expression of Jaws2 in the mediodorsal thalamic region of animals injected in the PFC. A) Schematic representation of field-of-view for histological analysis. B-L) Neuronal expression of Jaws2 in the mediodorsal thalamic region of animals included in the analyses. Yellow dotted outlines indicate subnuclei of the mediodorsal thalamic region, including central mediodorsal (MDC), medial mediodorsal (MDM), intermediate mediodorsal (IMD), paraventricular (PV) and centromedial (CM) thalamic regions.

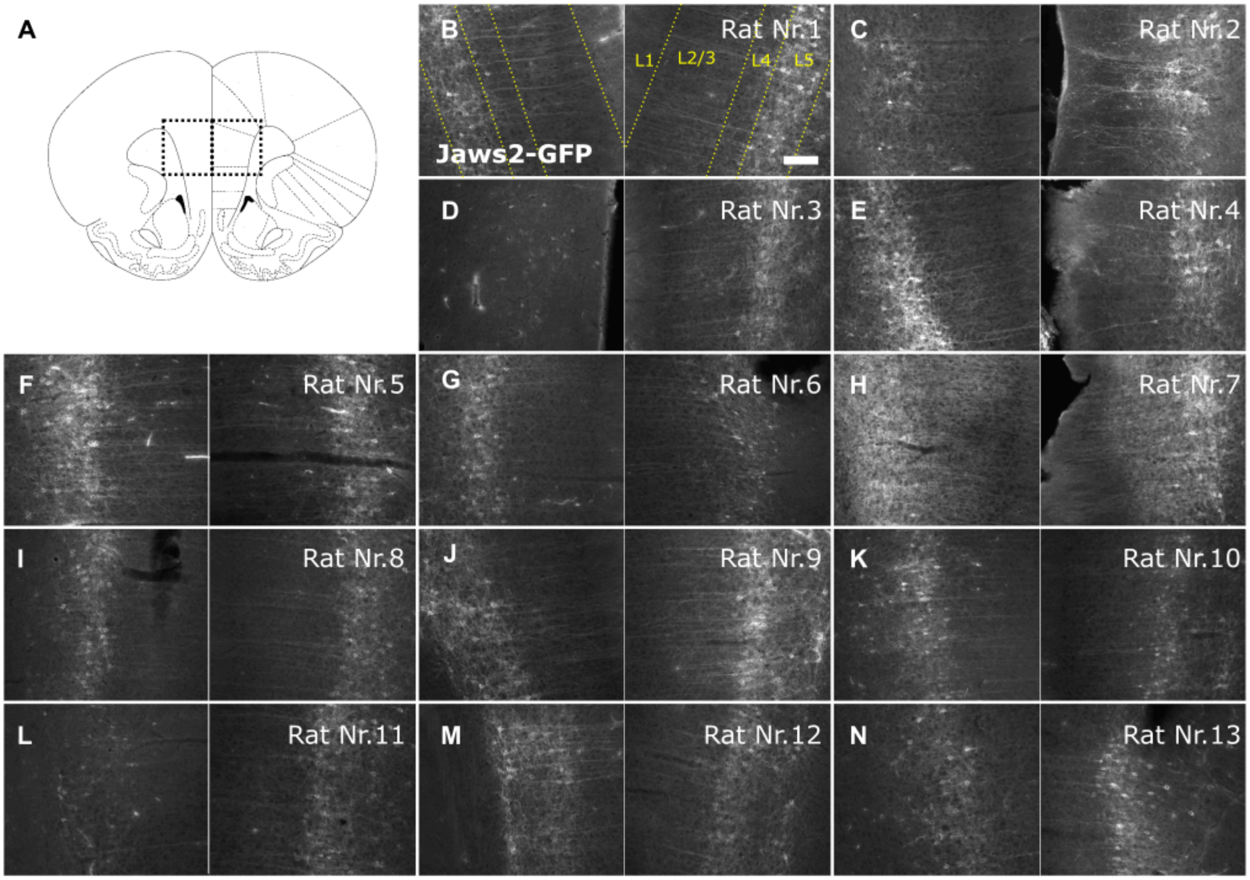

Supplementary Figure 2. Histological verification of the expression of Jaws2 in the prefrontal cortex of animals injected in the mediodorsal thalamus. A) Schematic representation of field-of-view for histological analysis. B-N) Neuronal expression of Jaws2 in PFC of animals included in the analyses. Yellow dotted outlines indicate PFC layer 1-5.

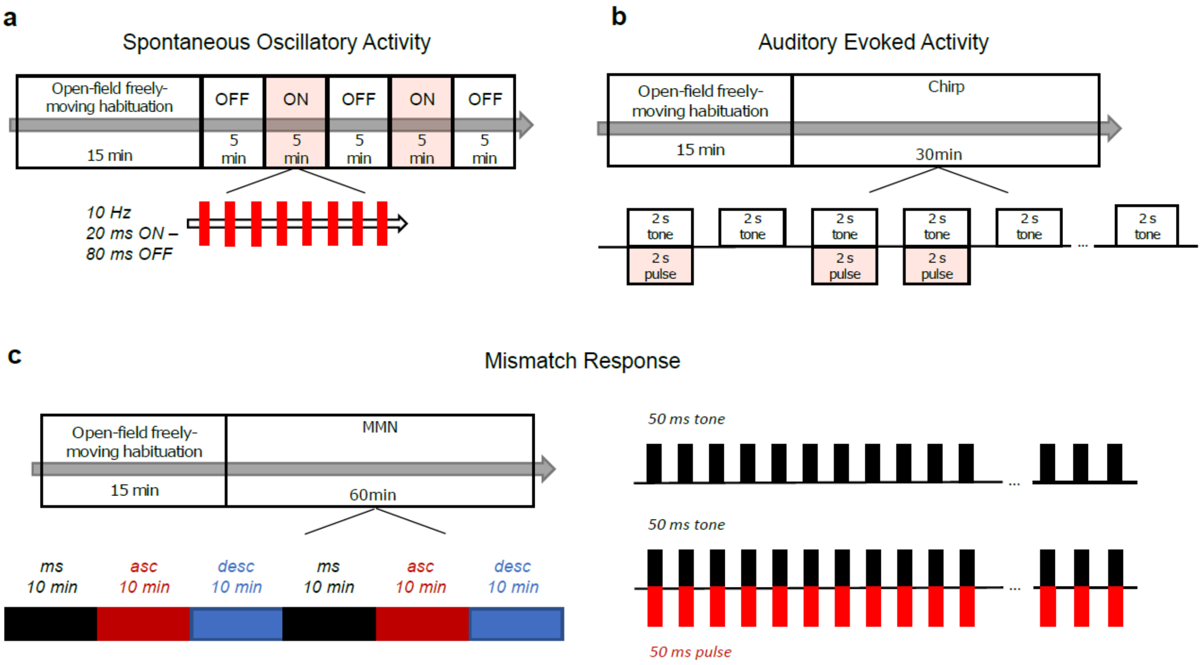

Supplementary Figure 3. Experimental design and paradigms. A) Quantitative electroencephalography (qEEG) to assess spontaneous oscillatory activity and coherence consisted of 15 minutes of freely-moving habituation followed by five 5-min-long periods of alternating 10 Hz photoinhibition (20 ms ON - 80 ms OFF) and no-photoinhibition. B) Auditory chirp-evoked potentials to infer auditory-evoked oscillatory activity were obtained through 15 minutes of freely-moving habituation followed by 30 minutes of chirp-modulated tones (300 trials) in which at random half of the trials were accompanied by 2 second-long LED continuous pulses. C) Mismatch negativity-like responses (MMN) were obtained through 15 minutes of freely-moving habituation followed by six 10 minutes-long sequences. Three types of sequences (each repeated twice) were used: i) many-standard (ms), ii) ascending (asc), and iii) descending oddball (desc) tone sequences. In each sequence, the target tone (e.g. 7 kHz) is in a different context: as a control, standard, or deviant tone. The paradigm was repeated twice: 1) without any photoinhibition, and 2) accompanying each tone with a 50 ms continuous LED pulse.

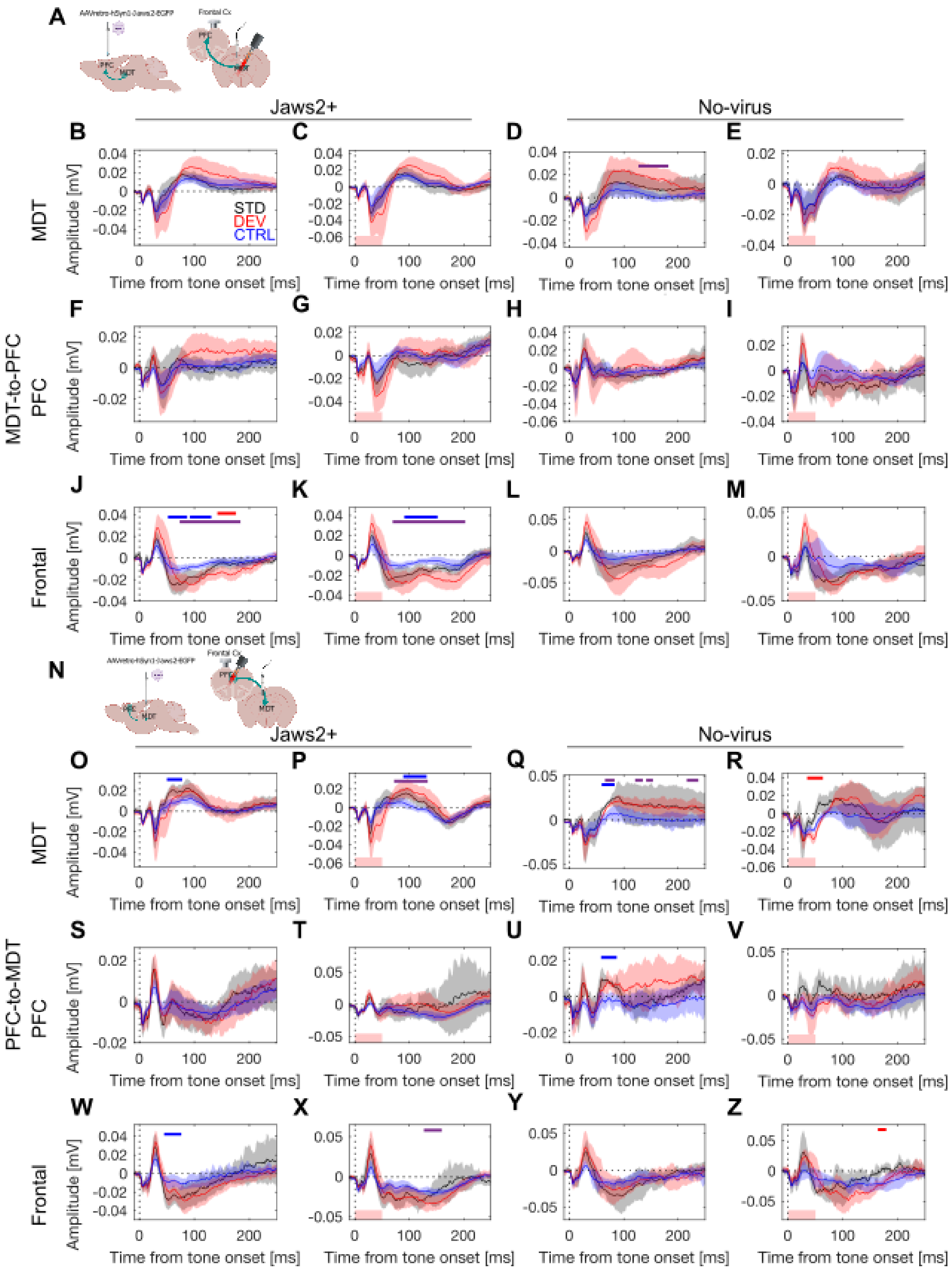
A
Frontal Cx
PFC
MDT
Jaws2+
No-virus
B
C
D
E
MDT
Amplitude [mV]
STD
DEV
CTRL
Time from tone onset [ms]
F
G
H
I
MDT-to-PFC
PFC
J
K
L
M
Frontal
N
O
P
Q
R
S
T
U
V
PFC-to-MDT
W
X
Y
Z

Supplementary Figure 4. Photoinhibition of the reciprocal PFC-MDT connection does not interfere with mismatch responses. A) Schematic of the MDT-to-PFC experiment. Average ERP waveforms for the MDT-to-PFC in B) LED-off (N=10) and C) LED-on (N=10) conditions in Jaws2+ animals, and D) LED-off (N=5) and E) LED-on (N=5) conditions in no-virus control animals in the MDT; average ERP waveforms for the F) LED-off (N=10) and G) LED-on (N=10) conditions in Jaws2+ animals, and H) LED-off (N=5) and I) LED-on (N=5) conditions in no-virus control animals in the PFC; and average ERP waveforms for the J) LED-off (N=10) and K) LED-on (N=10) conditions in Jaws2+ animals, and L) LED-off (N=5) and M) LED-on (N=5) conditions in no-virus control animals in the frontal cortex. N) Schematic of the PFC-to-MDT experiment. Average ERP waveforms for the PFC-to-MDT in O) LED-off (N=11) and P) LED-on (N=11) conditions in Jaws2+ animals, and Q) LED-off (N=4) and R) LED-on (N=4) conditions in no-virus control animals in the MDT; average ERP waveforms for the S) LED-off (N=11) and T) LED-on (N=11) conditions in Jaws2+ animals, and U) LED-off (N=4) and V) LED-on (N=4) conditions in no-virus control animals in the PFC; and average ERP waveforms for the W) LED-off (N=11) and X) LED-on (N=11) conditions in Jaws2+ animals, and Y) LED-off (N=4) and Z) LED-on (N=4) conditions 1in no-virus control animals in the frontal cortex. Evoked responses to the deviant (DEV, red); to the standard (STD, blue); and the control tone (CON; black). Solid lines indicate the average and shaded areas describe the standard error of the mean. Top horizontal lines indicate clusters identified by paired cluster-based permutation analysis. Red boxes at the bottom indicate LED-on stimulus duration (0-50 ms).
